# Using Implementation Research to Promote the Expansion and Sustainability of Intergenerational Programs

**DOI:** 10.1093/geroni/igaa057.2602

**Published:** 2020-12-16

**Authors:** Leslie Hasche

## Abstract

Implementation studies provide guidance on how to reduce the gap between empirically-supported interventions and routine care. For intergenerational programs that bring older adults and other generations together to promote social connection, improve health and well being, and to reduce ageism, the role of implementation science is rarely studied. Intergenerational programs have a long-standing role in social service and housing contexts, yet the quality of the evidence and sustainability of these programs is often in question. This symposium aims to demonstrate how implementation studies can identify available evidence and influential contextual factors to examine issues of adoption, fidelity, and sustainability of intergenerational programs. First, a scoping review of the available evidence on best practices of intergenerational programs will help highlight fidelity issues. Second, an environmental scan for intergenerational housing will highlight how contextual factors may impact the adoption and spread of intergenerational programs. Third, a pre-implementation study delivering multi-modal best practices training to local community sites will share indicators of the feasibility of training staff to implement evidence-based intergenerational practices. Finally, an evaluation of a community collaborative of organizations implementing intergenerational programs will highlight the process by which organizations develop and sustain partnerships. The chair will summarize how the studies’ methodological approaches incorporate implementation science and outcomes. Implications for both future research on organizational context, funding, and implementation strategies, as well as for practice settings will be named. The discussion will identify implementation gaps that will need to be overcome to expand and sustain intergenerational programs.

